# Global Distribution of Novel Rhinovirus Genotype

**DOI:** 10.3201/eid1406.080271

**Published:** 2008-06

**Authors:** Thomas Briese, Neil Renwick, Marietjie Venter, Richard G. Jarman, Dhrubaa Ghosh, Sophie Köndgen, Sanjaya K. Shrestha, A. Mette Hoegh, Inmaculada Casas, Edgard Valerie Adjogoua, Chantal Akoua-Koffi, Khin Saw Myint, David T. Williams, Glenys Chidlow, Ria van den Berg, Cristina Calvo, Orienka Koch, Gustavo Palacios, Vishal Kapoor, Joseph Villari, Samuel R. Dominguez, Kathryn V. Holmes, Gerry Harnett, David Smith, John S. Mackenzie, Heinz Ellerbrok, Brunhilde Schweiger, Kristian Schønning, Mandeep S. Chadha, Fabian H. Leendertz, A.C. Mishra, Robert V. Gibbons, Edward C. Holmes, W. Ian Lipkin

**Affiliations:** * Columbia University, New York, New York, USA; † University of Pretoria/NHLS Tswhane Academic Division, Pretoria, South Africa; ‡ Armed Forces Research Institute of Medical Sciences, Bangkok, Thailand; § National Institute of Virology, Pune, India; ¶ Robert Koch-Institut, Berlin, Germany; # Walter Reed AFRIMS Research Unit Nepal, Katmandu, Nepal; ** Hvidovre University Hospital, Hvidovre, Denmark; †† Centro Nacional de Microbiologia, Instituto de Salud Carlos III, Majadahonda, Madrid, Spain; ‡‡ Institut Pasteur Côte d’Ivoire, Abidjan, Côte d’Ivoire; §§ Curtin University of Technology, Perth, Western Australia, Australia; ¶¶ PathWest Laboratory, Nedlands, Western Australia, Australia; ## Severo-Ochoa Hospital, Leganés, Madrid, Spain; *** University of Colorado Denver School of Medicine, Aurora, Colorado, USA; ††† Pennsylvania State University, University Park, Pennsylvania, USA; ‡‡‡ National Institutes of Health, Bethesda, Maryland, USA

**Keywords:** picornavirus, rhinovirus, HRV-C, multiplex MassTag PCR, lower respiratory tract infection, childhood pneumonia, dispatch

## Abstract

Global surveillance for a novel rhinovirus genotype indicated its association with community outbreaks and pediatric respiratory disease in Africa, Asia, Australia, Europe, and North America. Molecular dating indicates that these viruses have been circulating for at least 250 years.

Acute respiratory illness (ARI) is the most frequent infectious disease of humans. Ordinary upper respiratory tract infections are usually self-limited; nevertheless, they result in major economic impact through loss of productivity and strain on healthcare systems. Lower respiratory tract infections (LRTIs) are among the leading causes of death in children <5 years of age worldwide, particularly in resource-poor regions (*1*). *Streptococcus pneumoniae* and *Haemophilus influenzae* are important bacterial causes of ARI, although their impact is expected to decline with increasing vaccine coverage. Collectively, however, viruses dominate as causative agents in ARI. Viruses frequently implicated in ARI include influenza virus, respiratory syncytial virus, metapneumovirus, parainfluenza virus, human enterovirus (HEV), and human rhinovirus (HRV).

HRVs are grouped taxonomically into *Human rhinovirus A* (HRV-A) and *Human rhinovirus B* (HRV-B), 2 species within the family *Picornaviridae* (International Committee on Taxonomy of Viruses database [ICTVdb]; http://phene.cpmc.columbia.edu). These nonenveloped, positive-sense, single-stranded RNA viruses have been classified serologically and on the basis of antiviral susceptibility profile, nucleotide sequence relatedness, and receptor usage (*2*). Phylogenetic analyses of viral protein VP4/VP2 and VP1 coding regions indicate the presence of 74 serotypes in genetic group A and 25 serotypes in genetic group B (*2*).

Isolated in the 1950s from persons with upper respiratory tract symptoms (*2*,*3*), HRVs have become known as the common cold virus because they are implicated in ≈50% of upper respiratory tract infections (*4*). Large community surveys, including the Virus Watch studies of the 1960–1970s (*5*), have shed light on some aspects of HRV biology and epidemiology. HRVs were also observed in LRTIs soon after their recognition (3), and data supporting a causative association have accumulated over the past decade (*6*,*7*). HRVs have also been implicated in exacerbations of asthma and chronic bronchitis and are increasingly reported in LRTIs of infants, elderly persons, and immunocompromised patients (*4*).

## The Study

The advent of broad-range molecular assays, including multiplex PCR and microarray systems, promises new insights into the epidemiology and pathogenesis of respiratory disease (*8*,*9*), given that a laboratory diagnosis is not routinely achieved for a substantial portion of respiratory specimens from symptomatic patients. We recently described the application of a multiplex PCR method for microbial surveillance wherein primers are attached to tags of varying mass that serve as digital signatures for their genetic targets. Tags are cleaved from primers and recorded by mass spectroscopy, enabling a sensitive, inexpensive, and highly multiplexed microbial detection. We used the multiplex MassTag PCR system (*10*) to investigate respiratory samples that had tested negative during routine diagnostic assessment. This previous study yielded pathogen candidates in approximately one third of cases, and in 8 cases identified a novel genetic clade of picornaviruses divergent from the previously characterized clades, including HRV-A and-B (*8*). To assess whether this novel clade circulates outside New York state, where it was discovered in cases of influenzalike illness (ILI), we investigated respiratory specimens from Africa, Asia, Australia, and Europe. In most studies (Africa, Asia), the collecting laboratories performed MassTag PCR, and inert mass-tagged amplification products were sent for analysis by mass spectrometry (MS); in other instances (Europe, Australia), inactivated nasopharyngeal swabs or aspirates were sent to New York for MassTag PCR and MS analysis.

Samples in South Africa were collected through a program for comprehensive surveillance of causes of respiratory illness in hospitalized children in the Pretoria area. MassTag PCR was applied to 58 specimens collected during the 2006 season from symptomatic children in their first year of life with no diagnosis available from previous clinical laboratory evaluation. Analysis of amplification products by MS yielded positive signal for HEV/HRV in 14 (24%) samples. Independent amplification and sequence analysis of VP4/2 coding sequence (*8*) in both laboratories showed sequences that matched the novel genotype in 4 (29%) samples obtained from patients with LRTIs and respiratory distress (Table 1, Figure). Samples collected in Côte d’Ivoire, West Africa, were from symptomatic persons living in the vicinity of Taϊ National Park. This location was the most remote of our study; residents have limited contact with other human populations. In this location, 2 (10%) HRV-A were identified in the 52 samples available for analysis (Table 1, Figure).

**Table 1:** Molecular diagnosis of ARI from 7 countries by using MassTag PCR* and VP4/2 sequencing†

Country	Season(s)	Samples	Picornavirus positive						
			Total	Novel clade	HRV-A	HRV-B	HEV	% Male	Age range (mean/median)
South Africa	2006	58	14	4	6	3	1	71	0.4–30 mo (5.6/3)
Côte d’Ivoire	2006	52	2	0	2	0	0	100	22–28 y (25/25)
Nepal	2005–06	80	17	4	7	5	1	56	0.25–56 y (8.5/3)
India	2007	50	6	3	3	0	0	83	4–36 mo (17.8/18)
Australia	2006	2	2	1	1	0	0	100	4–6 mo (5/5)
Denmark	2007	70	7	5	1	0	1	57	1–8 mo (2.9/2)
Spain	2003–2006	14‡	14	6	5	3	0	86	1–96 mo (23.2/15.5)

*See (*10*). †ARI, acute respiratory illness; HRV, human rhinovirus; HEV, human enterovirus. ‡With previous HRV diagnosis.

**Figure:**
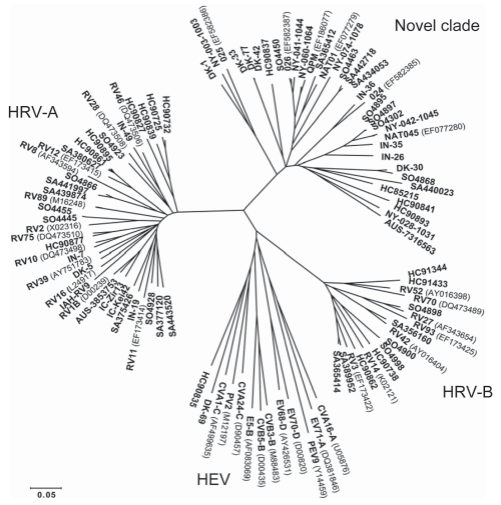
Phylogenetic analysis of VP4/2 coding region of viruses identified in association with acute respiratory illness (ARI) in South Africa, Côte d’Ivoire, Nepal, India, Western Australia, Denmark, and Spain (sequences deposited in GenBank under accession nos. EU697825–83). Phylogeny of VP4/2 nucleotide sequence (401 nt) was reconstructed by neighbor-joining analysis applying a Jukes-Cantor model; the scale bar indicates nucleotide substitutions per site. Included for reference are sequences belonging to the novel genotype identified in New York State (NY-003, –028, –041, –042, –060, and –074 [*8*]), similar viruses reported recently (QPM [*11*]; NAT01 and NAT045 [*12*]; and 024, 025, 026 [*13*]), and selected human rhinovirus A (HRV-A) serotypes (GenBank accession numbers for reference sequences are indicated in parentheses); HRV-B serotypes; human enterovirus C (HEV-C) viruses human coxsackievirus A1 and A24 (CV-A1, and CV-A24, respectively); human poliovirus 2 (PV-2); HEV-B viruses human echovirus 5 (E-5), human coxsackievirus B3 (CV-B3), and swine vesicular disease virus (CV-B5); HEV-D viruses human enterovirus 68 and 70 (EV-68, EV-70); porcine enterovirus B virus porcine enterovirus 9 (PEV-9); and HEV-A viruses human coxsackievirus A16 (CV-A16) and human enterovirus 71 (EV-71). SA, South Africa; IC, Côte d’Ivoire; HC, Nepal; IN, India; AUS, Australia; DK, Denmark; SO, Spain.

In Nepal, viruses of the novel genotype were identified in specimens collected during ILI surveillance or outbreaks of respiratory disease. Samples from ILI surveillance activities were collected in Kathmandu and Bharatpur. Outbreak samples were collected in the summer months from camps of >100,000 refugees from Bhutan located in Jhapa, southeast Nepal. Samples represented all age groups and were collected from December 2005 through July 2006. The novel genotype was identified by independent molecular typing in both laboratories in 4 (5%) samples (Table 1, Figure). In India, samples from 50 children with ARI, submitted for routine laboratory analysis during the 2007 season, were evaluated by MassTag PCR. Independent molecular typing in both laboratories indicated the novel genotype in 3 (6%) samples (Table 1, Figure).

Additional sample sets were obtained through main diagnostic laboratories in Western Australia, Denmark, and Spain, representing random respiratory specimens submitted for laboratory analysis. In 1 sample available from Western Australia, the novel genotype was identified in a preterm infant with undiagnosed, wheezy LRTI. The novel genotype was also found in 5 (7%) of 70 samples from Denmark and in 6 (43%) of 14 samples with previously diagnosed HRV infection from Spain (Table 1, Figure).

The 5% overall frequency of the novel genotype across our study samples, representing 34% of all detected picornavirus infections, and its observed global distribution, led us to analyze the accumulating sequence data for insights into their history. Rates of evolutionary change and the Time to the Most Recent Common Ancestor (TMRCA) of the novel clade were estimated by using the Bayesian Markov Chain Monte Carlo approach (BEAST package [*14*]; ), applying a relaxed molecular clock with an uncorrelated lognormal distribution of rates, a GTR + I + Γ_4_ model of nucleotide substitution (determined by MODELTEST [*15*]), and exponential population growth. Statistical uncertainty in each parameter estimate is expressed as 95% highest probability density (HPD) values. The estimated mean rate of evolutionary change was 6.6 × 10^–4^ substitutions/site/y (95% HPD = 0.3–14.6 × 10^–4^ substitutions/site/y; 38 dated samples collected over 32 mo (*8**,**16*) (S.R. Dominguez et al., unpub. data). Under this rate the mean TMRCA was estimated at 1,800 y, although with wide variance caused by the short sequence available (95% HPD = 279–5,201 y). Despite the inherent sampling error, this analysis suggests that this third clade of rhinovirus has been circulating for >250 years. The diversity observed within the novel clade and its genetic distance from other HRV/HEV were comparable to those seen for HRV-A, -B, or the HEV species (Table 2).

**Table 2:** Percentage of intraspecies and interspecies conservation of VP4/2 nucleotide sequence*

Viruses	HEV-A	HEV-B	HEV-C	PV†	HEV-D	HRV-A	HRV-B	New clade
HEV-A	72	61	63	63	63	59	61	60
HEV-B		75	64	64	59	59	61	59
HEV-C			75	71	62	61	65	61
PV				81	60	60	62	61
HEV-D					83	59	61	61
HRV-A						80	61	63
HRV-B							80	60
New clade								75

*HEV, human entero virus; PV, poliovirus; HRV, human rhinovirus. †PV may be moved by the International Committee on Taxonomy of Viruses into HEV-C.

## Conclusions

A clade of picornaviruses recently discovered in New York State is globally distributed and is found in association with community outbreaks of ARI and severe LRTIs of infants. These viruses contribute both to a substantial proportion of previously undiagnosed respiratory illness and to diagnosed, but nontyped cases of HRV infection. Similar viruses were recently characterized also in Queensland, Australia (*11*); California, USA (*12*); Hong Kong Special Administrative Region, People’s Republic of China (*13*); and Germany (*16*). Our findings indicate the need for further investigation into this third (HRV-C) group of rhinoviruses with emphasis on epidemiology, pathogenesis, and strategies to prevent and ameliorate disease caused by HRV infection.
